# Key Factors to Consider in Team Meetings when Dealing with Multimorbidity in Primary Care: Results from a Delphi Panel

**DOI:** 10.7759/cureus.4990

**Published:** 2019-06-25

**Authors:** Filipe Prazeres, Jose A Simoes

**Affiliations:** 1 Department of Medical Sciences, University of Beira Interior, Faculty of Health Sciences, Covilhã, PRT

**Keywords:** delphi technique, group meetings, group processes, multimorbidity, portugal, primary health care

## Abstract

Background: Multimorbidity brings several difficulties and challenges to the daily work of primary care teams. Team meetings are opportunities to discuss approaches and solutions on how to best manage multimorbid patients.

Objective: This qualitative study aimed to collect a consensus, from general practitioners that deal with multimorbid patients, about their perspectives regarding multimorbidity team meetings in primary care.

Methods: The study followed a modified Delphi method with 15 Portuguese general practitioners. After every round of responses, results were analyzed, and justifications for non-consensual items were aggregated by the investigators, and then a new Delphi round with the revised questionnaire was again initiated. This process was repeated until consensus has been reached.

Results: Overall, a list of 10 key themes associated with the ideal meeting was agreed: (a) definition; (b) setting; (c) duration; (d) frequency; (e) number of participants; (f) attendance; (g) requirement of patient’s presence; (h) number of patients/clinical cases; (i) structure of the meeting; and (j) sharing meeting results. The consensus was achieved after two Delphi rounds with a mean score between 7.9 and 8.7 (maximum score of 9.0 per key theme).

Conclusion: The complexity of multimorbidity affects meetings’ periodicity, duration, and participants. Ideally, it should be an interprofessional primary care team meeting. Further research exploring meeting outcomes (organizational effectiveness and healthcare quality) of the proposed factors is needed before they can be recommended for general use.

## Introduction

Primary care teams cannot evade dealing with multimorbid patients (i.e., patients with two or more chronic health problems, where none is an index condition) since they are very common in primary care settings [1-3]. This scenario is expected to worsen with the aging population and the prevalence increase of unhealthy lifestyle risk factors [2,4]. In people aged 65 years and older, multimorbidity may have a prevalence higher than 95% [5].

Multimorbidity in Portugal affects more than one-third of the population, and in adult primary care patients its prevalence increases up to 72.7% [6-7]. As predictable, such a high prevalence and disease burden bring several difficulties and challenges to the daily work of general practitioners (GPs), and the primary care team as a whole [8].

Previous results regarding GPs perspectives stated that GPs need teamwork and coordination with other healthcare professionals, families, and caregivers in order to manage the difficulties and challenges of multimorbidity [8]. It is also commonly accepted that more than single disease-oriented programs, person-centered approaches are necessary tools to manage the complexity of multimorbidity [4].

Team meetings may be the opportunities to discuss approaches and solutions on how to best manage multimorbid patients individually and also discuss the clinical cases that the primary care team wants to share uncertainties or reservations [9].

Managing meetings is not only an important skill to future physician leaders or academics but it is also linked to quality and safety in health care [10]. Meetings are said to be effective when decisions made are understood and well executed [11].

The purpose of this study was to collect a consensus, from general practitioners that deal with multimorbid patients, about their perspectives regarding team meetings in primary care in the context of multimorbidity.

## Materials and methods

The current study followed a modified Delphi method [12-14]. The Delphi technique is a data gathering and analysis method that aims “to seek out information which may generate a consensus on the part of the respondent group” (p.1), through an anonymous structured and iterative way [12].

Study design and data collection were approved by the Ethics Committee at the University of Beira Interior no. CE-UBI-Pj-2018-028. Completion and return of the questionnaires were regarded as informed consent.

Preparation phase

The investigators selected eight Portuguese GPs with a PhD (5 males, 3 females), distributed throughout the national territory based on being involved in primary care practice, research, and teaching activities to answer an open-ended questionnaire through email. Selected GPs were academic leaders in primary care.

The open-ended questionnaire consisted of one question that had to be answered in a free-text form: “How should a primary care team meeting be like in the context of multimorbidity? (i.e., what is the ideal meeting for healthcare teams dealing with multiple chronic disease patients at primary care settings?)”. Ten answer items were sought: (a) definition of team meeting; (b) ideal setting; (c) duration in minutes; (d) frequency of meetings; (e) number of participants; (f) who should attend? different professional groups?; (g) should the patient be present?; (h) the number of patients/clinical cases to be addressed per meeting?; (i) what is the structure of the meeting? who should preside, etc...?; and (j) with whom should the results of the meeting be shared?.

Investigators then converted the collected information of the 10 items into a structured questionnaire to be used in the modified Delphi study.

Modified Delphi study

For the Delphi panel, the investigators invited Portuguese GPs with and without PhD. The panel had to be consisted of at least 10-15 GPs and its members had to have experience in (i) treating multimorbid patients in ambulatory care; (ii) publication records in primary care; and (iii) management role in healthcare units [12].

The structured questionnaire was sent to the Delphi panel members, who were asked to express their opinion on each of the 10 items using a Likert-type ordinal scale of 1 to 9 (1=absolutely no agreement and 9 = full agreement). As in previous Delphi studies, the existence of consensus was defined by at least 70% of the panel members rating 7 or above each item. Ratings below 7 had to be justified [14].

After every round of responses, results were analyzed, and justifications for non-consensual items were aggregated by the investigators, and then a new Delphi round with the revised questionnaire was again initiated. This process was repeated until consensus has been reached.

The modified Delphi was conducted between September 2018 and January 2019.

Descriptive statistics (measures of central tendency and level of dispersion) were used to analyze the existence of consensus between Delphi rounds.

## Results

Fifteen of the 27 invited GPs accepted to participate in first Delphi round and in the second Delphi round (55.6% acceptance rate). Delphi panel members’ characteristics are given in Table 1.

 

**Table 1:** Delphi panel characteristics (n=15)

	n (%)	Mean (SD)
Sex		
Women	7 (46.7)	
Men	8 (53.3)	
Age (years)		49.0 (15.1) min=33; max=70
Experience in primary care (years)		19.7 (14.0) min = 5; max = 38
No. of patients with multimorbidity consulted per week		39.3 (21.6) min=15; max=75
No. of publications		23.7 (35.0) min=0; max=120
Management role in healthcare units (years)		4.33 (6.0) min=0; max=20

Table 2 shows that all items were consensual in round 1; at least 70% of the panel members rated 7 or above each item. Nonetheless, the investigators decided to do a second round with the revised questionnaire. In the second round, not only consensus increased but the mean ratings per item also increased. Table 3 displays the final consensus.

 

**Table 2:** Delphi rounds 1 and 2 (scores)

	1st Delphi Round		2nd Delphi Round	
Items	Consensus (7 or above) (%)	Mean (SD) ratings	Consensus (7 or above) (%)	Mean (SD) ratings
I	93.3	8.0 (0.93)	93.3	8.3 (0.88)
Ii	100	8.7 (0.59)	100	8.7 (0.46)
Iii	80	7.7 (1.45)	93.3	8.2 (1.01)
Iv	73.3	7.3 (1.40)	93.3	7.9 (1.39)
V	73.3	7.7 (2.19)	93.3	8.4 (0.91)
Vi	73.3	7.3 (1.58)	93.3	8.3 (0.96)
Vii	80	7.5 (2.17)	93.3	8.5 (0.92)
Viii	86.7	7.4 (2.32)	100	8.6 (0.74)
Ix	86.7	7.6 (1.99)	100	8.2 (0.78)
x	80	7.5 (2.13)	100	8.4 (0.74)

**Table 3:** Final consensus

Items	“How should a primary care team meeting be like in the context of multimorbidity?”
I- definition of team meeting	Periodic gathering of different professionals of the Health Unit who provide care for multimorbid patients; for transdisciplinary discussion and adoption of clinical and/ or organizational decisions; carried out in a predetermined time and place; with or without external guests. The agenda is well defined and previously known. One of the team members chairs the meeting.
II - ideal setting	Health Unit’s meeting room (or another room with appropriate conditions).
III - duration in minutes	Ideally less than 60 minutes. It can be variable, depending on the team's previous knowledge of the clinical cases and their complexity. It should not exceed 120 minutes in length.
IV - frequency of meetings	Every two weeks. Depending on the number and complexity of the multimorbidity present in the patients, another periodicity may be defined, not exceeding one-month interval. Warning: periodicity may change due to the competing need for discussion of other issues, organization, and maturity of the health team.
V - number of participants	All the necessary players, considering the capacity of the room.
VI - who should attend? different professional groups?	Family physicians should always be present. Depending on the nature of the problems involved, other health professionals should also be present: hospital doctors, nurses, social worker, psychologist, physiotherapist, pharmacist, and nutritionist.
VII - should the patient be present?	Normally not, except if absolutely necessary to expose the clinical case, or if the estimated treatment burden imposes the need for the patient's presence to decide therapeutic options.
VIII - number of patients/clinical cases to be addressed per meeting?	Due to the complexity of the multimorbid patient, approach up to two clinical cases per meeting. The number of clinical cases to be addressed per meeting will vary greatly depending on the team's experience in dealing with multimorbidity and the frequency and duration of meetings.
IX - what is the structure of the meeting? who should preside, etc...?	A chairman of these meetings should be appointed to identify, with the professionals of the Health Unit, the clinical cases that deserve broad discussion; and leads the meeting. Each case should be presented by the family doctor or nurse, listing difficulties/doubts in their management, followed by discussion and final definition of the consensus interventions. A facilitator is assigned. Other professional records what will be done to the patient.
X - with whom should the results of the meeting be shared?	The results of the meeting regarding the management of the patient(s) should be shared with all care providers in an effective and tailored way for each health professional, the patients, or their caregiver. Warning: Patient/Caregiver must previously consent to information sharing.

## Discussion

In the present study, a list of 10 factors associated with the ideal meeting of primary care teams dealing with multimorbid patients was agreed upon following a Delphi technique.

As expected, the current results suggest that it should be a “same time/same place” decision-making meeting, as it is the most common meeting type [10,15]. Also, it should have an agenda that was previously prepared and distributed (including clear purpose or goals); some authors consider this to be one of the most important aspects of a meeting [9,16].

Consistent with the literature, the role of the chairperson extends beyond running the meeting [10,17]. He is not only the team member that leads the meeting but also the person that plans it. Delphi participants agreed that the chairperson should in advance identify with the other healthcare professionals the multimorbid cases that need to be discussed. A note-taker will document the decisions taken during the meeting. After, clinical decisions should be shared with all care providers, the multimorbid patients, and their caregivers.

In accordance with the present results, previous authors have mentioned the importance of the capacity of the room (not crowded or too small) and the presence of the necessary equipment for a meeting to be effective [18].

The current study supports evidence from previous observations, family physicians need to work with other health professionals to better manage multimorbid patients (interprofessional primary care team meetings)[8].

As mentioned in the literature, a meeting should take at least one hour [16]. In the current study, the length of meetings was considered to vary from less than 1 hour to no more than 2 hours, depending on the complexity of the multimorbid cases planned to be discussed. The complexity of multimorbidity may modify not only the length of meetings but also their frequency and number of patients discussed (with an ideal of two clinical cases per meeting).

This study has several practical implications. Meetings of primary care teams dealing with multimorbid patients should follow traditional strategies for effective meetings---have a coordinator, a structure, and be held in an appropriate meeting room---as acknowledged in previous studies conducted in primary care settings [19]. Meetings should also be patient-centered with the complexity of patients’ multimorbidity adjusting the meetings’ periodicity, duration, and participants (interprofessional collaboration). However, the Delphi panel also decided that the presence of the patient during the meeting should be an exception; previous results showed that from the patients’ perspective it is important to participate or be represented, in the meetings [20].

The current study has some limitations. A small sample of GPs was used. Although they were carefully chosen, and the minimum number and good diversity of sample characteristics reached, caution must be applied, as the findings might not be generalizable to other settings. Further research with other health professionals dealing with multimorbid patients is needed to investigate the current topic from other perspectives (including the multimorbid patient’s perspective).

## Conclusions

The current study provides primary care teams with a set of aspects, relevant to health professionals dealing with multimorbid patients, to help better plan interprofessional team meetings. Overall, a list of 10 key themes associated with the ideal meeting was agreed: (a) definition; (b) setting; (c) duration; (d) frequency; (e) number of participants; (f) attendance; (g) requirement of patient’s presence; (h) number of patients/clinical cases; (i) structure of the meeting; and (j) sharing of meeting results. The consensus was achieved after two Delphi rounds with a mean score between 7.9 and 8.7 (maximum score of 9.0 per key theme). A primary care team meeting, in the context of multimorbidity, should be an every two weeks gathering of different professionals of the Health Unit who provide care for multimorbid patients. The meeting takes place in the Health Unit's meeting room for less than 60 minutes; should always include family physicians and all the necessary players to discuss up to two clinical cases. Normally the patient should not be present (except if necessary), and the meeting results should be shared with all care providers, the patients, or their caregivers. A chairman, a facilitator, and a note-taker should be appointed. Further research exploring meeting outcomes (organizational effectiveness and healthcare quality) of the proposed aspects are needed before they can be recommended for general use.
